# Biomarkers in Peripartum Cardiomyopathy—What We Know and What Is Still to Be Found

**DOI:** 10.3390/biom14010103

**Published:** 2024-01-12

**Authors:** Karolina E. Kryczka, Marcin Demkow, Zofia Dzielińska

**Affiliations:** Department of Coronary and Structural Heart Diseases, National Institute of Cardiology, 04-628 Warsaw, Poland

**Keywords:** peripartum cardiomyopathy, heart disease in pregnancy, heart failure, prolactin, 16-kDa PRL, cardiac biomarkers, fibrosis, galectin-3, myeloperoxidase, microRNA, heat shock protein, Hsp

## Abstract

Peripartum cardiomyopathy (PPCM) is a form of heart failure, often severe, that occurs in previously healthy women at the end of their pregnancy or in the first few months after delivery. In PPCM, the recovery of heart function reaches 45–50%. However, the all-cause mortality in long-term observation remains high, reaching 20% irrespective of recovery status. The incidence of PPCM is increasing globally; therefore, effort is required to clarify the pathophysiological background of the disease, as well as to discover specific diagnostic and prognostic biomarkers. The etiology of the disease remains unclear, including oxidative stress; inflammation; hormonal disturbances; endothelial, microcirculatory, cardiomyocyte and extracellular matrix dysfunction; fibrosis; and genetic mutations. Currently, antiangiogenic 16-kDa prolactin (PRL), cleaved from standard 23-kDa PRL in the case of unbalanced oxidative stress, is recognized as the main trigger of the disease. In addition, 16-kDa PRL causes damage to cardiomyocytes, acting via microRNA-146a secreted from endothelial cells as a cause of the NF-κβ pathway. Bromocriptine, which inhibits the secretion of PRL from the pituitary gland, is now the only specific treatment for PPCM. Many different phenotypes of the disease, as well as cases of non-responders to bromocriptine treatment, indicate other pathophysiological pathways that need further investigation. Biomarkers in PPCM are not well established. There is a deficiency in specific diagnostic biomarkers. Pro-brain-type natriuretic peptide (BNP) and N-terminal BNP are the best, however unspecific, diagnostic biomarkers of heart failure at the moment. Therefore, more efforts should be engaged in investigating more specific biomolecules of a diagnostic and prognostic manner such as 16-kDa PRL, galectin-3, myeloperoxidase, or soluble Fms-like tyrosine kinase-1/placental growth factor ratio. In this review, we present the current state of knowledge and future directions of exploring PPCM pathophysiology, including microRNA and heat shock proteins, which may improve diagnosis, treatment monitoring, and the development of specific treatment strategies, and consequently improve patients’ prognosis and outcome.

## 1. Introduction

Peripartum cardiomyopathy (PPCM) is a life-threatening disease leading to a deterioration in the systolic function of the left ventricle (LV) associated with pregnancy. The onset of heart failure (HF) is usually observed a few weeks before or in the first few months after delivery in women with no previous cardiac history [1]. PPCM is almost always characterized by a left ventricular ejection fraction (LVEF) under 45% on echocardiography with or without LV enlargement [2,3].

Cardiomyopathies are diseases of the heart muscle that include dilated, hypertrophic, restrictive, and arrhythmogenic cardiomyopathies and PPCM [4]. Recently, a new type of cardiomyopathy with a preserved LVEF has been distinguished [4]. The pathophysiology and morphology of different types of cardiomyopathies have been elucidated elsewhere [5]. Most types of cardiomyopathies differ morphologically and are easy to differentiate through echocardiography or cardiac magnetic resonance imaging (CMRI). PPCM may mimic dilated cardiomyopathy (DCM) in the case of a reduced LVEF with dilated LV. However, DCM usually occurs later in life due to the slow and irreversible process of heart muscle damage [4]. In the case of genetic mutations, an overlapping phenomenon may be observed, which means that different cardiomyopathies may share mutations in the same gene [5]. This phenomenon is observed in PPCM and DCM, most frequently in the titin gene (*TTN*) [5]. PPCM is characterized by an approximately 45–50% recovery rate, depending on the studied population, and a wide range of phenotypes and courses of the disease [1,2,6]. However, the mortality from PPCM remains high in long-term observation, and in some registries even reached 24% within three years of observation [2,7]. PPCM may relapse in future pregnancies, especially in patients who did not improve an LVEF of ≥50% [2,7]. Even in patients who recovered from PPCM, the subsequent pregnancies may again decrease LV contractility. Although the deterioration of LV function associated with subsequent pregnancy is greater in PPCM patients who do not show improved LV function, the mortality rate during eight years of observation is similar, reaching 20% irrespective of the value of the LVEF before subsequent pregnancies [8].

**Figure 1:**
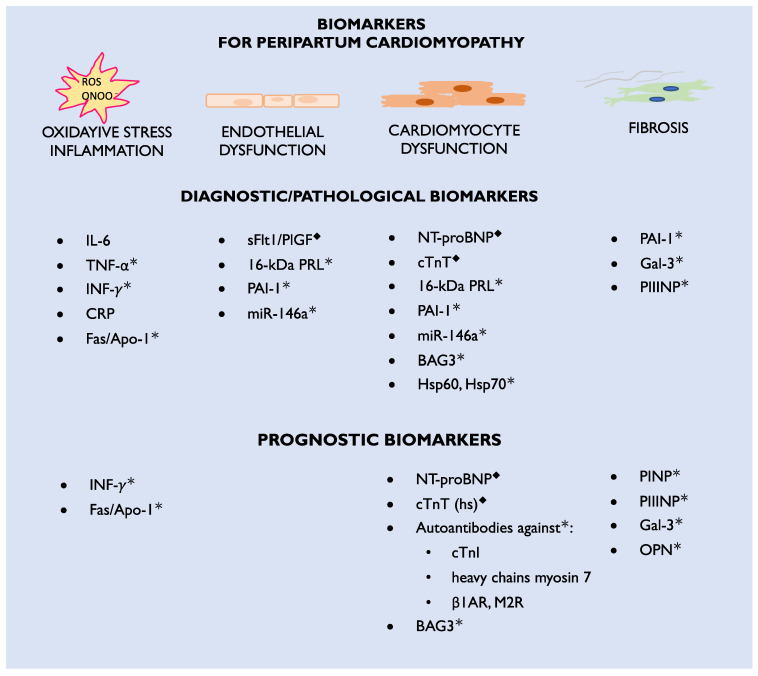
Diagnostic and prognostic biomarkers for peripartum cardiomyopathy; β1AR—beta 1-adrenergic receptors; CRP—C-reactive protein; cTnI—cardiac troponin I; cTnT (hs)—cardiac troponin T (high specific); Fas/Apo1—apoptosis antigen-1; Gal-3—galectin-3; Hsp—heat shock protein; IL-6—interleukin-6; INF-γ—interferon gamma; M2R—M2-muscarinic receptors; miR—microRNA; NT-proBNP—N-terminal pro-Brain-type natriuretic peptide; ONOO^•^—peroxynitrite; OPN—osteopontin; PAI-1—plasminogen activator inhibitor-1; PINP—procollagen type-I N-terminal propeptide; PIIINP—procollagen type-III N-terminal propeptide; PlGF—placental growth factor; PRL—prolactin; ROS—reactive oxygen species; sFlt1—soluble Fms-like tyrosine kinase-1; TNF-α—tumor necrosis factor alpha; ^◆^—biomarkers currently used in clinical practice; *—biomarkers which are candidates for future clinical practice.

The etiology of the disease is complex and not fully recognized, including unbalanced oxidative stress leading to the formation of 16-kDa prolactin (PRL) with antiangiogenic and cardiotoxic properties. Bromocriptine, which blocks PRL being released from the pituitary gland, is currently the most specific PPCM treatment. However, not all patients respond to this treatment. This may be due to other mechanisms beyond 16-kDa PRL or delayed diagnosis. Therefore, novel pathophysiological pathways and biomarkers need further examination, particularly those engaged in microcirculatory, cardiac muscle, and extracellular matrix dysfunction. Currently, there is a deficiency in specific diagnostic and prognostic biomarkers that can be widely used in clinical practice to distinguish the symptoms observed in physiological pregnancy and puerperium from those pathological signs associated with PPCM. According to the International Programme on Chemical Safety, on behalf of the World Health Organization, biomarkers are defined as “any substance, structure, or process that can be measured in the body or its products and influence or predict the incidence of outcome or disease” [9]. This definition indicates the investigation of a broad range of body tissues and genes. Most of the biomarkers already known to be associated with PPCM may be classified according to their role in the pathophysiology of the disease or their diagnostic and prognostic utility (Figure 1). In this review, we present the current state of the knowledge on PPCM’s pathophysiology and the biomarkers already used in clinical practice, as well as new biomarker candidates, and new scientific directions which may improve the diagnosis and outcome of this frequently life-threatening disease.

### 1.1. Epidemiology, Risk Factors, and Outcomes

As PPCM is a rare disease, the sources of data on different biomarkers are limited. Moreover, the study population’s number of participants did not exceeded 151 patients. It is not uncommon for a certain biomarker to be investigated in only one study with a limited number of patients. For this review, studies available from online medical databases on the topic of biomarkers in PPCM were used. In addition, some representative case reports were presented to highlight new ideas.

The exact and up-to-date statistics on PPCM epidemiology are limited. So far, it has been reported that the disease is most frequent in Nigeria (1:100 deliveries), Haiti (1:300 deliveries), and South Africa (1:1000 deliveries). In the United States, among Caucasians, the frequency increased from 1:2500 in 2004 to 1:1316 deliveries in 2011 [10,11,12,13,14,15]. This process is associated with the older age of and concomitant diseases affecting mothers, such as hypertension or diabetes [1,16]. The data from the six-month observation of PPCM women in the EuroObservationRegistry Project indicate that PPCM occurs globally, and the frequency in Europe may be comparable to that in Africa [17].

The risk factors for PPCM include the mother’s older or younger age (>30 years old and <18 years old, respectively), multiparity, twin pregnancies, hypertension, preeclampsia, smoking, diabetes, and race [1,2]. The disease may be underdiagnosed, since signs of HF usually mimic those associated with normal pregnancy and puerperium, such as fatigue or leg edema. Data from the registry validated the main risk factors for PPCM, with preeclampsia being observed in almost one-fourth of patients. The registry highlighted the importance of not only relying on physical examination, as over 40% of PPCM patients did not present with peripheral edema or pulmonary congestion [6,17].

In most patients, the onset of PPCM was mainly observed in the first month postpartum with sever impairment of LVEF, <35%. The mortality of mothers and neonates was high, reaching 6% and 5%, respectively. In mothers, sudden cardiac death was the main cause of death. The frequency of thrombosis and rehospitalization reached 7 and 10%, respectively. LVEF recovery was observed in less than half of the patients, calling for the improvement of treatment [6].

### 1.2. Different Phenotypes and Courses of Peripartum Cardiomyopathy

As mentioned before, the disease may affect women with a broad range of risk factors, requiring different types of treatment. To illustrate this, here we present two examples of different courses of the disease with as assessment of their biomarkers.

**Figure 2:**
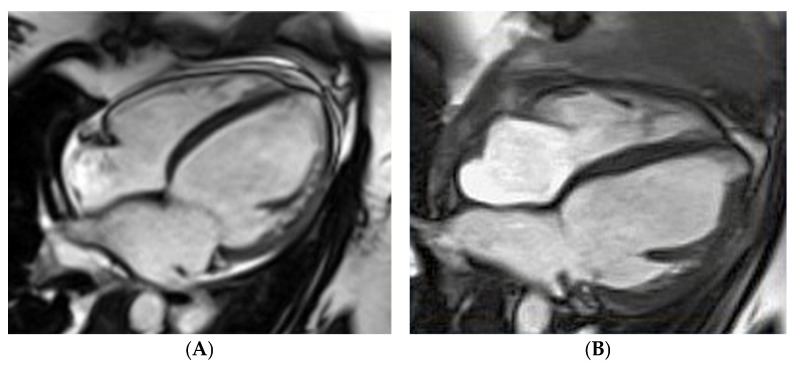
Peripartum cardiomyopathy in a gravida para26-year-old woman in cardiac magnetic resonance imaging: (**A**) fourth day postpartum, left ventricular ejection fraction (LVEF) of 17%, (**B**) after bi-ventricle assist device treatment, LVEF of 35% [18].

The first case concerns the acute onset of HF in a 26-year-old woman on the third day after delivery of her first pregnancy. Figure 2 demonstrates an enlarged LV with an LV end-diastolic diameter of 68 mm and a severe decrease in LVEF, up to 17%, assessed by CMRI. Apart from pharmacological treatment, also with bromocriptine, the patient required interventional treatment with a biventricular assist device (BiVAD). As a result, the LVEF increased to 35% with BiVAD treatment (Figure 2B) and further to 40–45% in the six-month follow-up period [18]. The effect of the treatment was monitored with biomarkers. NT-proBNP decreased approximately 10 times during treatment, from baseline 10,275 pg/mL (N < 125) to 1019 pg/mL at the six-month observation. Cardiac troponin T was 52.78 ng/L (N < 14.00) at baseline and 7.18 after six months.

**Figure 3:**
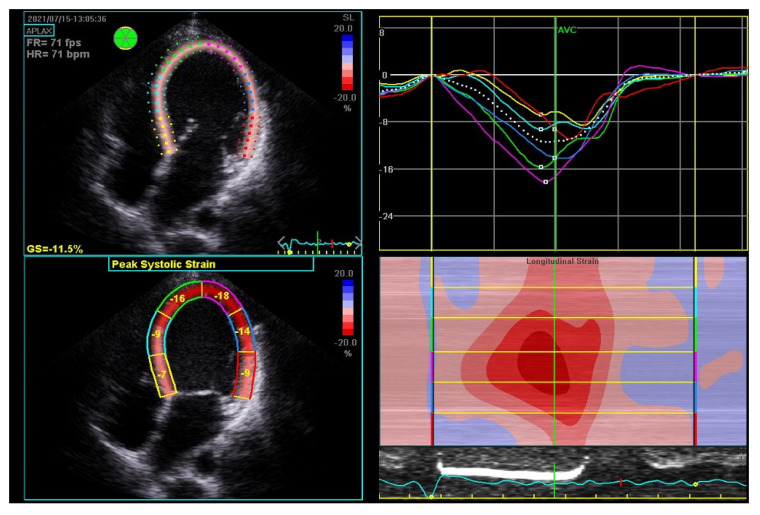
Global strain in echocardiography of 35-year-old woman with peripartum cardiomyopathy with severe impairment of left ventricular (LV) heart muscle functions. Four-chamber apical view: diastolic (**upper left**); systolic (**bottom left**); LV systolic and level and asynchrony of maximal contractility of LV segments illustrated by lines of different colors (**upper right**); longitudinal strain map of LV (**bottom right**); FR—frame rate, fps—frames per second; GS—global strain longitudinal; HR—heart rate; bpm—beats per minute [19].

By contrast, we hospitalized a 35-year-old woman on the fourth day after her third cesarean section, who presented with atrial fibrillation, a severe decrease in LVEF to 20%, an enlarged LV end-diastolic diameter of 62 mm, and severe mitral insufficiency. The global longitudinal strain (GLS) was impaired up to −11.5% (norm from −26% to −18%) (Figure 3). According to the literature, a GLS > −11.4% is recognized as a predictor of increased cardiovascular events, death, and the lack of improvement of LVEF ≥50% [19]. The LV insufficiency was still present after successful cardioversion. The pharmacological treatment of HF with bromocriptine was introduced, and the patient showed an improved LVEF of over 50% at the six-month observation. The biomarkers were significantly elevated from the baseline: NT-proBNP equaled 6776.00 pg/mL (N < 125.00) and cardiac troponin T 33.38 ng/L (N < 14.00). The biomarkers’ levels decreased during the six-month follow-up to 170.40 pg/mL for NT-proBNP and to 4.99 for TnT.

## 2. Pathophysiology

### 2.1. Oxidative Stress and Inflammation, Endothelial and Microcirculatory Dysfunction

PPCM may mimic DCM; however, advanced examination has revealed substantial differences between these cardiomyopathies. Ultrastructural analysis of the heart muscle has demonstrated that the loss of filaments and widened spaces between myofibrils were comparable in PPCM and DCM patients [20]. However, ultrastructural signs of advanced dysfunction in microcirculation were detected only in the case of PPCM, including structural alterations to endothelial cells and apoptotic bodies in myocardial microcirculation, which were cleaved by macrophages. Endothelial dysfunction with apoptotic bodies blocking capillaries distinguishes PPCM from DCM and myocarditis. Additionally, heart muscle biopsies from PPCM patients, but not those from DCM patients, revealed the presence of preadipocytes, known for their ability to transform into endothelial cells [20]. Other differences include the presence of activated mast cells that mediate the microvascular inflammatory response. This finding may partially elucidate the ability of self-restoring cardiac function observed in PPCM.

**Figure 4:**
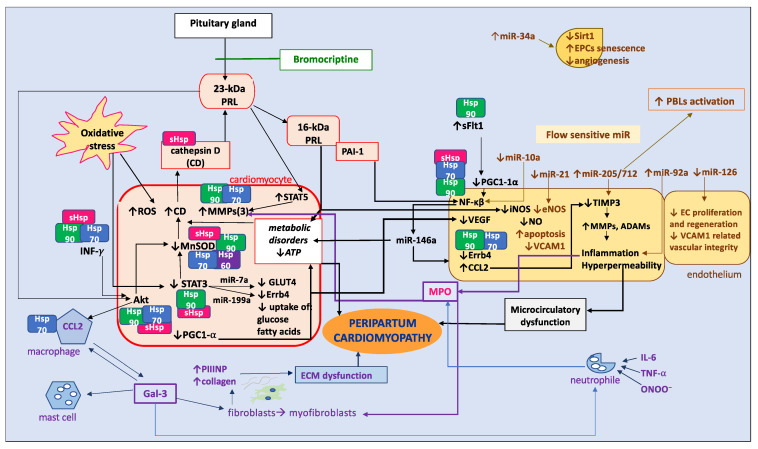
Pathophysiology of peripartum cardiomyopathy (PPCM), changed based on the results of the paper by Hilfiker-Kleiner et al. [21]. Rose background highlights main pathophysiological pathways of unbalanced oxidative stress triggering 16-kDa prolactin-related heart tissue injury; blue-violet pathway presents extracellular matrix (ECM) fibrogenesis; yellow-brown pathway shows microRNAs in PPCM, their discovered and hypothetical roles; navy, green, and pink squares represent heat shock proteins and their presence in PPCM pathophysiological pathways; ADAMs—ADAM metalloproteinases; ATP—adenosine triphosphate; β-AR—beta adrenergic receptor; CCL2—CC chemokine ligand-2; CD—cathepsin D; EC—endothelial cell; eNOS—endothelial nitric oxide synthase; EPCs—endothelial progenitor cells; Errb4—erb-B2 receptor tyrosine kinase-4; Gal-3—galectin-3; GLUT4—glucose transporter type 4; Hsp—heat shock protein; IL-6—interleukin-6; INF-γ—interferon gamma; iNOS—inducible nitric oxide synthase; miR—microRNA; MMP3—metalloproteinase-3; MnSOD—manganese superoxide dismutase; MPO—myeloperoxidase; NF-κβ—nuclear factor kappa beta; NO—nitric oxide; ONOO^−^—peroxynitrite; PAI-1—plasminogen activator inhibitor-1; PBLs—peripheral blood leucocytes; PGC1-1α—peroxisome proliferator-activated receptor-γ coactivator-1α; PIIINP—procollagen type-III N-terminal propeptide; PlGF—placental growth factor; PRL—prolactin; ROS—reactive oxygen species; sFlt1—soluble Fms-like tyrosine kinase-1; Sirt-1—sirtuina-1; STAT—signal transducers and activators of transcription; TIMP-3—tissue inhibitor metalloproteinase-3; TNF-α—tumor necrosis factor alpha; VCAM—vascular cell adhesion molecule; VEGF—vascular endothelial growth factor.

PPCM pathophysiology is complex and not fully recognized (Figure 4). It includes oxidative stress, inflammation, and microcirculatory and cardiac tissue dysfunction, as well as hormonal and genetic backgrounds [1,21]. According to the current knowledge, the main trigger of the disease is unbalanced oxidative stress that, in case of decreased antioxidative mechanisms, mainly signals the transducers and activators of transcription (STAT) 3 and leads to the enhanced activation of cathepsin D. In a high-PRL-level environment in the heart tissue, this leads to increased cleavage of the regular 23-kDa PRL chain to antiangiogenic 16-kDa PRL [21]. On the one hand, 16-kDa PRL enhances endothelial dysfunction, and on the other hand, disturbs the metabolism of cardiomyocytes [21,22]. The great majority of these effects are mediated by miR-146a, whose production is stimulated via the nuclear factor kappa beta (NF-κβ), and activated by complexes of 16-kDa PRL and plasminogen activator inhibitor-1 (PAI-1). Secreted in exosomes and absorbed by cardiomyocytes, miR-146a triggers metabolic disorders by decreasing the expression of glucose transporter type 4 (GLUT4) and glucose uptake. In the endothelium, miR-146a decreases cell proliferation and promotes apoptosis, causing angiogenesis imbalance [22]. Independently from miR-146a, 16-kDa PRL causes endothelial dysfunction by inducing caspase-dependent apoptosis, as well as decreasing the synthesis of inducible nitric oxide synthase (iNOS) and, subsequently, nitric oxide (NO) production [21].

Endothelial dysfunction may also be caused by an increased level of soluble Fms-like tyrosine kinase-1 (sFlt1), which decreases the bioavailability of endothelial growth factors, especially the placental growth factor (PlGF). The level of sFlt1 increases with age [23]. sFlt-1 was found to induce HF in cardiac-specific cardiac peroxisome proliferator-activated receptor-γ coactivator-1α heterozygous knockout (HKO) mice. It was 5–10-fold elevated in women with PPCM 4–6 weeks postpartum compared with the controls (*p* = 0.002) [23]. In this group, preeclampsia, which is caused by increased sFlt-1 levels, was observed in almost one-third of PPCM patients compared with the general population rate of less than 10% [23]. High preeclampsia frequency in PPCM patients was validated by the EURObservational Research Programme PPCM registry [17]. Interestingly, in women with preeclampsia, higher levels of substrates for the production of vasoinhibins, such as PRL, placental lactogen, and growth hormone, as well as proteolytic enzymes secreted by the placenta, including cathepsin D, metalloproteinases (MMPs) MMP-2 and MMP-3, and bone morphogenic protein-1, were observed, compared with women in normal pregnancy. These resulted in higher levels of vasoinhibins in patients with preeclampsia, including 16-kDa PRL [24]. Therefore, it is suspected that vasoinhibins contribute to preeclampsia by increasing the risk of hypertension by decreasing placental neovascularization, vascular permeability, and dilatation [24]. The vasoinhibin-related mechanism is another possible pathway, beyond the sFlt-1/PlGF disturbances, that connects preeclampsia with PPCM. These data may elucidate why the time directly after delivery, when the concentration of vascular angiogenic peptides decreases, is the most vulnerable state for women to develop PPCM.

### 2.2. Heart Muscle Tissue

#### 2.2.1. Cardiomyocytes

As mentioned previously, the metabolism of cardiomyocytes in PPCM is severely disturbed. First, the deficiency in proper antioxidative mechanisms, mainly decreased STAT3, leads to decreased GLUT4 and erb-B2 receptor tyrosine kinase expression in cardiomyocytes and decreased glucose and fatty acid uptake [25]. Second, secreted in exosomes and absorbed by cardiomyocytes, miR-146a triggers metabolic disorders by decreasing GLUT4 expression and glucose uptake [22]. These changes cause decreased adenosine triphosphate production and decreased contractility, causing damage to cardiomyocytes. The earlier this destructive mechanism is interrupted, the less severe the deterioration in LV function and the greater the chance of LVEF recovery.

#### 2.2.2. Fibrosis

Other findings that partially elucidate the ability of the heart muscle to recover in PPCM are the limited area of detectable fibrosis in CMRI in the acute stage and the deficiency of fetal EH-myomesin expression. EH-myomesin is a cardiac protein expressed in fetal life. In DCM, EH-myomesin expression acts as a cardioprotective mechanism that prevents excessive and rapid deterioration of heart muscle function; however; at the same time; it limits its ability to recover [26].

Other factors that may enhance recovery from PPCM while acting directly on heart muscle tissue are sFlt-1 and PlGF. In biopsies from PPCM patients, the level of sFlt-1 mRNA in cardiac tissue is higher than in patients with idiopathic dilated cardiomyopathy (iDCM) [26]. The concentration of PlGF mRNA in heart tissue is also higher in PPCM than in iDCM patients. In this case, the higher PlGF concentration in PPCM patients, both in serum and in cardiac tissue, may contribute to the approximately 50% recovery rate observed in this form of cardiomyopathy, indicating that microvascular dysfunction is one of the major underlying causes of PPCM and that a refractory response to the antiangiogenic environment is as a form of treatment. Additionally, sFlt-1 has been found to act protectively directly in the heart tissue. sFlt1 prevented HF in mice by inhibiting an increase in monocyte chemoattractant protein-1 production and, consequently, monocyte infiltration of heart tissue and fibrosis [27].

#### 2.2.3. Extracellular Matrix

PPCM’s initial presentation is often acute. Therefore, there are frequently no or limited areas of fibrosis detected in CMRI [28]. This partially elucidates the ability of the heart muscle to recover. Extracellular matrix (ECM) fibrogenesis should be considered as a third underappreciated component in the pathophysiology of PPCM. Nowadays, the ECM has started to be considered not only as a stiff scaffolding for cardiomyocytes but also as a dynamic network of cells and chemokines, and an environment for biochemical processes, cell division, and differentiation to occur in [29]. Alterations to the ECM’s architecture and function affect cardiomyocytes and, globally, heart tissue function. Therefore, proper exploration and understanding of these processes are essential for the complex treatment of heart diseases, including PPCM [29]. As demonstrated in Figure 4, in the case of inflammation, endothelial cells and neutrophils produce an increased level of myeloperoxidase (MPO), which induces MMPs, decreases tissue inhibitors of metalloproteinases (TIMPs), enhances proliferation and activation of fibroblasts, collagen synthesis, and fibrosis. Furthermore, activated macrophages synthesize higher levels of galectin-3 (Gal-3), which increases fibrosis and additionally activates mast cells, monocytes, and neutrophils, which in turn synthesize more MPO.

Although PPCM occurs globally, the causes and outcomes of the disease vary in different populations. The differences include the fibrosis process in the ECM, which was found to exist in German (G) and South African (SA) cohorts [30]. More profibrotic biomarker profiles were found in the SA-PPCM patients, including a lower procollagen type-I N-terminal propeptide to procollagen type-III N-terminal propeptide (PINP/PIIINP) ratio, compared with the representative controls. Although the SA-PPCM patients had a higher LVEF at the baseline than G-PPCM patients (30 ± 9% vs. 24 ± 8%, *p* < 0.05), the outcome was less favorable than in the G-PPCM patients (full recovery: 32% vs. 52%, *p* = 0.003; mortality: 14% vs. 0%, *p* < 0.05). These findings suggest that, in PPCM, a higher rate of fibrosis decreases the rate of recovery. In addition, the study highlighted the importance of intensified medical therapy. While 100% of the G-PPCM patients received HF therapy with a beta-blocker, angiotensin convertase enzyme inhibitor (ACE-I) or angiotensin receptor blocker (ARB), bromocriptine, and heparin, only 75% of the SA-PPCM patients were treated with a beta-blocker and ACE-I/ARB, while 26% received bromocriptine and 7% received an anticoagulant [30]. The predictors of unrecovered HF included a lower LVEF at baseline (in G-PPCM, the mean LVEF at baseline was 25% in non-recovered vs. 28% in recovered patients, while in SA-PPCM, 15% vs. 32%, *p* < 0.05). Higher levels of Gal-3, sST2, and OPN, and a lower PINP level and PINP/PIIINP ratio at baseline were also a risk factor for persistent LV dysfunction. The PIIINP levels did not differ significantly between these subgroups.

Increased PINP production is associated with reactive concentric heart muscle hypertrophy and reactive fibrosis, inflammation, and edema in the cardiomyocytes. On the other hand, PIIINP production leads to eccentric hypertrophy and increased fibrosis associated with increased heart muscle damage, and the death of cardiomyocytes [31,32]. Importantly, PINP was lower and PIIINP was higher in SA-PPCM than in G-PPCM. These findings indicate that, in G-PPCM, the healing reaction turned toward PINP production, while in SA-PPCM, it turned toward PIIINP production, increased fibrosis, and muscle damage. These observations align with previous findings that PINP increases more in patients with HF with preserved EF (HFpEF) than in patients with HF with reduced EF (HFrEF), indicating that the overproduction of collagen I is less harmful than that of collagen III [31,32]. In addition, in a group of 111 patients with decompensated HF, the patients with lower PINP levels had better outcomes than the patients with higher PINP serum levels, validating a better prognosis for patients without heart tissue fibrosis [32].

#### 2.2.4. Genetics

Genetic background is another significant risk factor in PPCM pathophysiology. Mutations associated with PPCM have been found in approximately 20% of patients [33]. The most frequent mutations were reported for the sarcomere protein titin (TTN) gene, which was observed in 10–15% of patients [33]. Many other mutations in PPCM are shared with DCM, including the desmoplakin (DSP) gene, the *LMNA* gene from the nuclear lamina, the *FLCN* gene which encodes the protein in intercalated discs, and the *BAG3* gene which encodes a co-chaperon of the heat shock protein Hsp70 [33]. This fact elucidates the family history of DCM in some patients with PPCM.

The most frequent truncating mutations in the *TTN* gene are known to have different levels of penetrance [34,35]. A lower disease penetrance was observed in women (62–82%) than in men, indicating that sex is an independent risk factor for cardiomyopathies. In addition, there was no difference in the recovery rate between patients with HF and *TTN* truncating mutations and patients with no *TTN* mutations [36]. The presence of this mutation can enable PPCM to occur; however, it does not have predictive value and is not associated with the outcome. Recovery from HF is possible in this group of patients. As the penetrance of the *TTN* mutation is not 100%, other predisposing factors must exist, either genetic or environmental. In the case of PPCM, the additional factors may include 16-kDa PRL, increased pre- or afterload, autoimmune processes, arrhythmia, or other chronic diseases, such as hypertension or diabetes [34,37]. This phenomenon may be defined as a second-hit concept, which assumes that a triggering factor influences the manifestation of a genetic mutation.

Newly reported mutations associated with PPCM include C825T polymorphism in the *GNB3* gene, related to poorer outcomes in PPCM patients, and single-nucleotide polymorphism in the parathyroid hormone-like hormone gene [38,39]. However, the roles of these variants must be evaluated in further studies. There are some implications of mutations in the *DSP* and *FLNC* genes. These mutations predispose patients to dangerous arrhythmias, and in some circumstances, an implantable cardioverter defibrillator may be considered [33]. *LMNA* mutations predispose patients to more severe HF than, for example, *TTN* truncating mutations [33]. Some data indicate overlapping mutations in PPCM and cancers, including mutations in DNA damage response pathway genes, such as *ATM*, *ERCC5*, *NBN*, *RECQL4*, and *SLX4* [5,40]. PPCM patients with these mutations have a 16-fold higher risk of developing cancer both before and after PPCM presentation [40]. These mutations predispose PPCM patients to heart muscle damage due to oxidative stress during pregnancy or chemotherapy for pre-existing cancer. Genetic mutations are one of the puzzles in the etiology of PPCM. The multifactorial hit concept assumes that the aggravation of different risk factors in a woman evokes PPCM [5].

## 3. Biomarkers

Currently, only one unspecific biomarker of HF is used in the diagnosis of PPCM: N-terminal pro-brain-type natriuretic peptide (NT-proBNP). Many more specific biomarkers, which are discussed in this review, are proposed for the diagnosis and prognosis of the disease course. However, most of them have not been validated or the laboratory detection process is complicated, precluding their use in everyday clinical practice [41,42].

### 3.1. Biomarkers Currently Used in Clinical Practice

#### 3.1.1. NT-proBNP and Brain-Type Natriuretic Peptide (BNP)

NT-proBNP and brain-type natriuretic peptide (BNP) are natriuretic peptides originating from the heart’s endocardium [43]. Their blood levels mainly increase due to mechanical stress evoked by fluid overload. NT-proBNP and BNP arise from proBNP as a result of the degradation by corin or furin [44]. BNP is biologically active, while NT-proBNP is inactive. The half-life of NT-proBNP is longer than BNP; therefore, its blood level is higher. BNP is a cardiac hormone that binds to natriuretic peptide receptor-A and activates cGMP pathways. BNP has a significant role in diuresis and natriuresis, vasodilatation, inhibition of the renin-angiotensin-aldosterone system, and modulation of the sympathetic nervous system [44]. NT-proBNP and BNP are clinically used unspecific biomarkers that cause an increase in HF of different etiologies as a result of unbalanced pressure and fluid overload [43]. BNP and NT-proBNP possess a high negative predictive value for blood levels of <128 pg/mL for NT-proBNP and <100 pg/mL for BNP [43]. These are the best diagnostic biomarkers of HF in pregnancy and after delivery at the moment, despite physiological changes during pregnancy that include fluid overload, an increased cardiac output, and glomerular filtration rate [43,45,46]. BNP and NT-proBNP norms in healthy pregnancy were defined recently in a study that included 260 healthy pregnant women sampled for BNP and NT-proBNP in every trimester [47]. The upper reference norms for NT-proBNP were established as 200 pg/mL in the first and second trimesters and as 150 pg/mL in the third trimester (the normal limit for young non-pregnant patients is 125 pg/mL). The levels of NT-proBNP in the third trimester had to be adjusted for body mass weight. In addition, the levels in the obese pregnant women were significantly lower during all pregnancy. Thus, in obese women, NT-proBNP values should always be adjusted for body weight. The upper norm for BNP was established at 50 pg/mL, with no differences based on the trimester [47].

NT-proBNP possesses a prognostic value. An analysis of 237 women with PPCM demonstrated that, in a subgroup of 110 PPCM patients with measured NT-proBNP levels, the patients who experienced adverse events, such as a composite endpoint of all-cause mortality, an extracorporeal membrane oxygenation, a ventricular assist device, or orthotopic heart transplantation in median 3.6 years (IQR 1.1–7.8) of observation, had a higher level of NT-proBNP (≥2585 pg/mL; log-rank test *p*-value 0.018) [48]. In women with cardiovascular diseases, performing several measurements throughout pregnancy is recommended to foresee the risk of cardiovascular events [43]. An increase in blood levels should trigger further cardiological evaluation with echocardiogram performance [43]. However, in obese patients, a low level of natriuretic peptides does not preclude an HF diagnosis, and therefore, should be used with caution [43].

#### 3.1.2. Cardiac Troponin T (cTnT) and Cardiac Troponin I (cTnI) 

Cardiac troponin T (cTnT) and cardiac troponin I (cTnI) are the main structural and functional proteins in cardiac sarcomeres, responsible for heart muscle contractility and relaxation. Together with troponin C, cardiac troponins form a complex attached to another protein, tropomyosin, which, together with actin, forms myofilaments. Myofilaments are the main part of the contractility apparatus in every kind of muscle in the body. Troponins T and I have different isoforms in the heart and skeletal muscles. Therefore, cardiac isoforms of these troponins serve as specific biomarkers of heart muscle damage, especially in acute coronary syndromes and acute or deteriorated HF of different origins, and also in pregnancy and puerperium, irrespective of the measurement method [43,49]. The release of cardiac troponins from cardiomyocytes is mainly caused by ischemia with the necrosis of cardiomyocytes but also by cardiomyocyte stretch, increased pressure and overload, short-term ischemia, and increased activity in the sympathoadrenal system [50]. The high specific (hs) troponin measurement method is preferable, as it enables the detection of lower levels of the biomarker and recognition of the condition earlier. As PPCM is a rare disease, not many studies address the cardiac troponin levels issue.

In one study that included 64 patients and 53 healthy postpartum controls, the median value of hs cTnT was only mildly elevated in the PPCM patients compared with the controls: 19 ng/L (9–699) vs. 2 ng/L (2–6), normal values < 4 ng/L, *p* < 0.0001 [51]. Another study revealed the prognostic value of hs-cTnT in PPCM [52]. In a group of 106 PPCM women, the patients with hs-cTnT levels of >4 ng/L at baseline had lower LVEF at the six-month follow-up than the patients with hs-TnT levels of ≤4 ng/L: 35.4% vs. 50.2%, *p* = 0.0001 [52]. No comprehensive studies were performed on cTnI. In some case reports, cTnI was elevated as well [53].

#### 3.1.3. Soluble Fms-like Tyrosine Kinase-1 and Placenta Growth Factor

sFlt-1 is a soluble form of Fms-like tyrosine kinase-1 (Flt-1) with a deficiency of transmembrane and intracellular domains. Flt-1 is a cellular receptor of the vascular endothelial growth factor (VEGF) and PlGF [23]. The soluble form, sFlt-1, is produced as a result of alternative splicing and, by binding to the circulating VEGF and PlGF, acts as a regulator of their circulating levels. As mentioned before, during pregnancy, sFlt-1 is also produced by the placenta and plays a significant role in the pathogenesis of PPCM and eclampsia with the diagnostic potential of the sFlt-1/PlGF ratio [23]. The serum level of PlGF was found to be higher in patients with PPCM compared with women after delivery (median [IQR] 97.5 [77.5–125.5] vs. 29 [19.2–40.8] ng/mL, respectively; *p* < 0.0001), as well as in non-pregnant women with acute HF (median [IQR] 98 (78–126) and 19 (16–22) ng/mL, respectively; *p* < 0.001; cut-off value threshold of 32 ng/mL) and comparable to healthy non-pregnant controls [41]. The serum level of sFlt-1 in PPCM patients was also higher than in healthy non-pregnant controls and comparable to non-pregnant patients with acute HF [41].

sFlt-1 is produced by the placenta. Therefore, its serum level is higher during pregnancy and at delivery than postpartum. At the same time, PlGF was lower in PPCM than in women in physiological pregnancy. As PPCM is mainly diagnosed after delivery, in this study, the serum level of sFlt-1 and PlGF in PPCM patients was lower than in physiological pregnancy and at delivery [41]. The sFlt-1/PlGF ratio was found to be significantly lower in patients with PPCM compared with physiological pregnancy and delivery. The ratio was also lower than in non-pregnant patients with acute HF (1.2 [0.9–2.8] and 9.8 [6.6–11.3], respectively; *p* < 0.001). Therefore, the authors of this study propose the sFlt-1/PlGF ratio as a diagnostic marker of PPCM diagnosed after delivery. Nothing is known about the sFlt-1/PlGF ratio in patients with PPCM diagnosed during pregnancy. The utility of the sFlt-1/PlGF ratio in PPCM diagnosis requires further evaluation by other clinical studies. For comparison, in patients with preeclampsia, the mean sFlt-1/PlGF ratio was 91.33 ng/mL, significantly higher than in physiological pregnancy (17.62 ng/mL). The best cut-off value for predicting preeclampsia was 24.96 ng/mL (sensitivity and specificity of 84.2 and 85.0%, respectively) [54]. However, in clinical settings, a cut-off value of 85 ng/mL is considered to foresee preeclampsia [55,56].

#### 3.1.4. 23-kDa PRL

23-kDa PRL is a protein hormone produced by the pituitary gland. 23-kDa PRL released to the blood regulates various physiological activities, such as mammary gland growth and milk production in women, progesterone synthesis in corpus luteum and secretion, the inhibition of estrogen synthesis in ovaria, ovulation, and the promotion of testosterone synthesis and secretion. In addition, 23-kDa PRL acts as a regulator of fluid and electrolyte balance with a decrease in sodium and potassium extraction, increased electrolyte absorption in the intestine, and water accumulation [57]. 23-kDa PRL is also produced by nervous system cells and via a paracrine and autocrine effect, regulating proliferation and differentiation processes [58]. The serum blood concentration of 23-kDa PRL increases throughout pregnancy and remains high while breastfeeding. As mentioned before, 23-kDa PRL, in case of uncontrolled oxidative stress, becomes a substrate for the production of pathologic 16-kDa PRL, which triggers PPCM [21]. Although 23-kDa PRL is not specific to PPCM, its level was found to be higher than in healthy gravida and age-matched peripartum controls: 24.7 vs. 7.4 ng/mL, *p* < 0.0001 [59]. PRL decreased significantly from 28.8 to 19.6 ng/mL, delta −9.23, *p* = 0.0068 in PPCM women who improved their LVEF in six months compared with non-improvers: from 27.65 to 21.5 ng/mL, delta −6.1 ng/mL, *p* = 0.45 [59]. The above data emphasize the prognostic potential of PRL. In clinical practice, it is possible to guide bromocriptine treatment by assessing the PRL blood level in the acute state and increasing the dose to 10–20 mg/day until the PRL concentration normalizes or prolonging the bromocriptine treatment over recommended eight weeks, regarding the clinical state of the PPCM patient and the PRL serum level [34,60].

### 3.2. Biomarker Candidates for Future Practice

#### 3.2.1. 16-kDa PRL

16-kDa PRL is a vasoinhibin, a NH_2_-terminal protein chain cleaved from 23-kDa PRL by cathepsin D [24]. The pathways of action of 16-kDa PRL are not clearly recognized, and study results are often contradictory [61]. 16-kDa PRL inhibits NO synthesis through iNOS downregulation in the endothelium. A study investigating the pathways in this process demonstrated that 16-kDa PRL alone acts by blocking the IFN regulatory factor-1 pathway, but not the NF-κβ signaling pathway, to the iNOS promoter [61].

16-kDa PRL was found to be highly generated from recombinant PRL in the supernatant of the LV heart tissue of PPCM conditional knockout (CKO) mice with an inactivated STAT 3 gene in cardiomyocytes, whereas no PRL cleavage was observed in the case of the healthy controls [21]. 16-kDa PRL was elevated in the serum of women with PPCM compared to healthy nulliparous women [42]; therefore, this biomarker is a good candidate for diagnostic purposes. However, the methodology for analyzing 16-kDa PRL is complex and, nowadays, is limited to scientific research [42].

#### 3.2.2. Gal-3

Gal-3 is a lectin from the carbohydrate-recognition domain that binds β-galactosides and possesses an additional proline- and glycine-rich N-terminal domain that enables it to form oligomers. Additionally, Gal-3 can interact with unglycosylated molecules via protein–protein interactions. Gal-3 is expressed in myeloid and epithelial cells; thus, it is present in many tissues and organs and is involved in a broad range of intra- and extracellular processes [62]. In cytosol, Gal-3 binds to several proteins of the apoptosis-signaling pathway, including Bcl-2, APO1-Fas, and Alix/AIP1. In nuclei, Gal-3 regulates gene transcription and is a pre-mRNA splicing factor [63]. Gal-3 is present on the surface of the cellular membrane and ECM, acting through cell–cell and cell–matrix interactions [63]. Gal-3 plays a crucial role in angiogenesis, inducing ECM fibrosis and the activation of fibroblasts, macrophages, and mast cells. Its increased level is associated with inflammation, apoptosis, and an increased risk of cardiovascular events, including HF and death [63]. However, to date, only two studies have investigated Gal-3 in PPCM [30,64]. In a group of 100 PPCM patients, the plasma level of Gal-3 was elevated compared with the healthy controls: 13.9 ± 3.7 vs. 8.5 ± 4.4, respectively; *p* < 0.0001. There was no association of Gal-3 levels with LVEF in the six- and twelve-month observations. However, in the PPCM patients who suffered from major cardiovascular events, the Gal-3 levels were higher. An increased level of Gal-3, especially in the third tertile, was a significant predictor of a higher cardiovascular event rate [65]. In the second study on the role of fibrosis in PPCM mentioned previously, Gal-3 was one of the factors associated with unrecovered heart muscle function [30]. More studies on Gal-3 in PPCM patients are required, as Gal-3 is perceived as a candidate for the therapeutic targeting of heart muscle remodeling [65].

#### 3.2.3. PINP and PIIINP 

PINP and PIIINP are the biomarkers of collagen synthesis. These molecules are produced during collagen I and III formation from procollagen, which is split by specific proteinases into N- and C-terminal propeptides released into the blood [31]. Like other markers of fibrogenesis, they require further investigation. More attention should be paid to procollagen type III N-terminal propeptide, which increases heart muscle stiffness, and as mentioned before, higher levels of it are associated with inferior cardiovascular outcomes irrespective of LVEF [30,32]. The ratio of procollagen types may possess additional diagnostic or prognostic value [30].

### 3.3. Markers of Inflammation

#### 3.3.1. Fas/Apo-1

Fas/Apo-1 is an apoptosis-signaling surface receptor that triggers programmed cell death in cardiac tissue. Fas/Apo-1, also called CD95, is a member of the tumor necrosis factor receptor superfamily. The Fas/Apo-1 receptor is activated via binding to the Fas membrane ligand. The Fas/Apo-1 transmembrane receptor may be released into circulation by the action of MMPs [66]. Plasma Fas/Apo-1 concentration was found to be elevated in HF of different etiologies and in Black PPCM patients compared with healthy controls (5.99 ± 4 U/mL vs. 0.84 ± 0.21 U/mL, *p* = 0.0003). Moreover, patients who died from PPCM had higher Fas/Apo-1 concentrations compared with PPCM women who survived (8.98 ± 4.5 vs. 5.33 ± 3 U/mL, respectively, *p* = 0.02) [67]. This study demonstrated the diagnostic and prognostic potential of this biomarker. However, due to the small study sample of 29 Black PPCM patients and 20 controls, this finding requires further investigation. The diagnostic and prognostic features of soluble Fas/Apo-1 were validated in a cohort of 100 PPCM patients and 20 controls [68]. The plasma Fas/Apo-1 level was significantly higher in the PPCM patients who died before the six months of observation were over than in the rest of the patients (9.6 [2–18.4] vs. 5.4 [0.4–14.9], *p* = 0.002) and was an independent predictor of death in multifactorial logistic regression analysis (OR = 3.56, CI 95% = 1.35–9.42).

#### 3.3.2. C-Reactive Protein (CRP)

C-reactive protein (CRP) is an inflammatory biomarker widely used in clinical practice. It is an unspecific marker, and depending on the study, the results are highly variable. It is synthetized in hepatocytes induced by pro-inflammatory cytokines, mainly interleukin-6 (IL-6) [69]. CRP has different forms, such as native pentamer (pCRP) or monomer (mCRP). Such forms bind to different receptors and lipid rafts, causing distinct biological effects. Native pCRP possesses both inflammatory and anti-inflammatory properties [69]. However, splitting it into mCRP gives it a solely proinflammatory influence on the endothelium, endothelial progenitor cells, lymphocytes, and platelets [69]. It inhibits endothelial NO synthase expression and induces endothelial cells to produce monocyte chemoattractant protein, IL-8, intracellular adhesion molecule-1, and vascular cell adhesion molecule-1. In addition, it inhibits neutrophil apoptosis, activates the classical component pathway, increases the procoagulant state by platelet activation and macrophage induction to tissue factor synthesis, and increases the expression of PAI-1 [69].

In one study, CRP was higher in the PPCM patients than in the controls: 10.8 ± 13.2 vs. 3.1 ± 0.9 mg/L, respectively; *p* < 0.01 [68]. The CRP plasma level at the baseline correlated positively with the LV end-diastolic and end-systolic diameters (rs = 0.33 and 0.35, respectively; *p* < 0.001). There was a negative correlation between the CRP level and the baseline LVEF (rs = −0.27, *p* = 0.015) [68]. However, there were no differences in CRP between survivors and non-survivors [68]. In another study, the CRP level in PPCM patients was even lower than in the postpartum controls: median 9 mg/L (range 0.5–180) vs. 38 mg/L (10–164), respectively; *p* < 0.01 [51].

#### 3.3.3. IL-6

IL-6 is a cytokine with many different effects on organisms, including inflammation, coagulation, antiapoptotic gene activation, cell proliferation, and differentiation. IL-6 binding to its transmembrane receptor activates STAT transcription factors, such as STAT 3, which promote cell survival and cell-cycle transition [70]. IL-6 increases the procoagulant state by inducing the synthesis of PAI-1 [70]. In addition, IL-6 possesses anti-inflammatory properties by inducing anti-inflammatory cytokine synthesis and inhibiting TNF-α production [70].

In one study, IL-6 was higher in PPCM women than in the healthy controls (7.9 ± 4 pg/mL vs. 1.4 ± 1.8 pg/mL, *p* < 0.0001). Moreover, IL-6 concentration was higher in PPCM patients with left ventricular thrombus compared with the rest of the PPCM patients (14.4 ± 7 pg/mL vs. 5.7 ± 4 pg/mL, *p* = 0.0004) [67]. In another study, IL-6 was not elevated in PPCM or unspecific for PPCM, similar to CRP [51]. The mean IL-6 level was insignificantly elevated compared with the postpartum controls (10.6 pg/mL ± 6.8 vs. 7.1 pg/mL ± 5.2, *p* = ns).

#### 3.3.4. Tumor Necrosis Factor Alpha (TNF-α)

Tumor necrosis factor alpha (TNF-α) is an inflammatory cytokine that influences heart muscle function. It is produced by cells in the immune system which are triggered by inflammation. In addition, TNF-α is produced within the myocardium as a response to many other forms of cardiac injury [71,72]. Physiological levels of TNF-α can protect the heart muscle against injury. However, higher levels of TNFα cause myocardial dysfunction and negative remodeling [72]. This is because TNF-α can mediate both pathways of cell growth and differentiation, as well as cell apoptosis and inflammation. Additionally, in a low-STAT 3 environment, the survivor activating factor enhancement pathway, initiated by TNF-α in case of a deterioration in blood circulation as protection from myocardial dysfunction, is insufficient [71,72]. TNF-α was found to be elevated in HF in DCM and PPCM patients [68,69,73]. In one study, TNF-α was a diagnostic biomarker but not a prognostic one, as there was no difference in the TNF-α serum levels of PPCM survivors vs. non-survivors [67]. In another study, pentoxifylline was found to decrease TNF-α levels in PPCM patients compared with standard HF treatment [73]. At the same time, the mortality rate was lower in the pentoxifylline-treated PPCM group (*n* = 1, 3.6% vs. *n* = 8, 32%) [73].

#### 3.3.5. Interferon Gamma (IFN-γ)

Interferon gamma (IFN-γ) is a cytokine that takes part in host defense in the immune system. It activates the STAT 1 transcription factor; induces inflammatory and cell-mediated immune responses, including the presentation of antigens to antigen presentation cells; activates natural killers; and regulates the activation of B cells and helper T lymphocytes [74,75]. IFN-γ activates macrophages that synthesize the chemoattractants of the immune cells at an inflammation site [76].

In the PPCM patients, IFN-γ was found to be significantly higher than in the controls: 2.9 vs. 0.16 IU/mL, *p* <0.001 [59]. Moreover, it was the only one of the inflammatory markers that decreased in the patients with improved LVEF but not in the non-improvers during the six-moth follow-up: 1.3 vs. 3.0 IU/mL, delta for improvers −1.87 vs. delta +0.86 for non-improvers, *p* = 0.018 [59]. The decrease in IFN-γ correlated with the decrease in the NT-proBNP level, confirming not only the diagnostic but also the prognostic manner of this biomarker. Delta IFN-γ correlated negatively with delta LVEF and positively with delta PRL [59].

#### 3.3.6. PAI-1

PAI-1 is a member of the fibrinolytic system and plays a significant role in the inhibition of fibrinolysis via the attenuation plasmin formation through the inhibition of both the tissue plasminogen activator and urinary plasminogen activator [77]. Plasma PAI-1 is synthesized by endothelial cells and hepatocytes and it is widely present in smooth muscle cells, fat cells, macrophages, lymphocytes, and platelets. PAI-1 takes part in many other processes, such as inflammation, atherosclerosis, and insulin resistance [77].

PAI-1 was increased in the PPCM patients at baseline compared with the healthy postpartum controls (64 ± 38 ng/mL vs. 16 ± 10 ng/mL, *p* < 0.01) [51]. The level of PAI-1 positively correlated with the level of miR-146a; however, it did not correlate with NT-proBNP or LVEF. The level of PAI-1 increased in the subsequent pregnancies of women with a history of PPCM before HF exacerbation. Therefore, PAI-1 may be considered a diagnostic but not a prognostic biomarker [51].

### 3.4. Autoantibodies to Cardiac Antigens

Autoantibodies against β1-adrenergic receptors (β1AR) and M2-muscarinic receptors (M2R) are frequently found in patients with DCM and hypertrophic cardiomyopathy (HCM) [78,79,80]. These antibodies bind to the second extracellular loop of the receptors on the membranes of cardiomyocytes and chronically activate them. This leads to cardiac dysfunction, apoptosis, damage, and, consequently, to HF [81]. In physiological processes, β1AR is activated by catecholamine–sympathetic nervous system mediators [82]. This activation increases contractility, cardiac output, and heart rate. M2R is activated by acetylcholine released by the parasympathetic nervous system. In a contradictory compensative manner, this activation leads to a decrease in cardiac contractility, output, and heart rate [83].

One study attempted to assess the role of antibodies against the cardiac sarcomeres’ heavy chain myosin 7 and cTnI in 70 PPCM patients in comparison with 50 healthy pregnant women [84]. The presence of at least one of these antibodies was reported in 46% of the patients with PPCM compared to 8% of the healthy controls. It was associated with lower baseline LVEF and a lower rate of recovery in the six-month follow-up period. In a second study, the frequency of autoantibodies acting against β1AR and M2R was higher in PPCM patients compared to healthy controls [85]. The presence of the autoantibodies mentioned above was validated in other etiologies of HF. The presence of these two types of receptors’ antigens was positively correlated with NT-proBNP, LV dimension, and NYHA class. At the same time, it conversely correlated with LVEF and LV fractional shortening. The presence of these antibodies increased the risk of PPCM onset (OR 18.8, *p* = 0.012). These findings partially elucidate the effectiveness of plasmapheresis beyond the removal of inflammatory mediators and toxic proteins, which was reported in two patients with severe PPCM [86]. However, the exact role of plasmapheresis in PPCM treatment requires further investigation.

## 4. New Specific Biomarkers to Be Found

### 4.1. Fibrosis and Inflammation

To improve the prediction and diagnosis of PPCM, efforts are being made to discover new specific biomarkers. The biomarkers that were not studied in PPCM or require further investigation are related to inflammation and fibrosis. One of them is myeloperoxidase (MPO), which is recognized as a biomarker of inflammation and oxidative stress [87,88,89]. Higher MPO levels are associated with inferior outcomes in cardiovascular diseases and a higher risk of death during one-year follow-ups (HR 1.51, *p* = 0.045) [90]. MPO is mainly released by neutrophils; however, it may be synthesized in endothelial cells, especially under inflammatory conditions. It increases MMP activity, decreases tissue inhibitors of MMPs, and induces fibrosis [87,88,89].

MMPs are a family of enzymes involved in ECM protein cleavage and may induce negative ventricular remodeling in cardiovascular diseases [31,91]. Several subgroups of MMPs are distinguished according to catalytic sites, aminoacidic chain similarity, and substrate affinity, such as collagenases, gelatinases, stromelysins, and metrilysins [92]. The hemopexin domain is crucial for collagen degradation, while the catalytic domain can cleave non-collagen substrates as well [91]. Increased levels of MMP-3 and MMP-2 have already been found in PPCM [31,59]. In other studies, increased levels of MMP-3 in HCM patients were associated with arrhythmias [92,93]. MMP-2 is a gelatinase that plays a crucial role in ECM remodeling [91]. It can be secreted by cardiomyocytes, fibroblasts, and endothelial and inflammatory cells. Importantly, MMP-2 degrades the proteins in the contractile apparatus: myosin light chain-1 and troponin I [91]. To date, the MMPs/TIMPs balance has not been analyzed in PPCM. TIMPs are an important part of the ECM homeostasis apparatus. Apart from the inhibition of MMPs, TIMPs have pleiotropic functions, including apoptosis inhibition, cell proliferation, and angiogenesis induced by VEGF [89,94]. Four known TIMPs are constantly expressed in normal heart tissue; however, the expression of TIMP-1 increases in pathological conditions [94]. Any disturbances in the MMP to TIMP ratio are thought to possess a pathological influence on heart tissue homeostasis.

### 4.2. MicroRNA

MicroRNAs (miRNAs or miRs) are small non-coding RNAs of approximately 22 kbp, which play a significant, mainly negative, role in the regulation of translation [95]. Therefore, miRNAs influence a great majority of physiological processes [96]. Some miRNAs have their pathological roles validated in HF [97]. However, to date, only one study has been conducted on the role of miRNAs in PPCM [22]. Halkein et al. studied the role of miRNAs associated with the NF-κβ pathway, which is an effector of 16-kDa PRL. Among a few studied miRNAs, miR-146a was found to play a significant role in PPCM, as mentioned previously [22]. New biomarker candidates may be found among a broad range of endothelial microRNAs, most of which are flow-sensitive and involved in the regulation of the endothelial function influencing the cell cycle, apoptosis, nitric oxide signaling, or inflammation. These miRs may be recognized as potential new pharmacotherapy targets. Figure 4. shows selected endothelial miRs that may be significant for treating PPCM by decreasing angiogenesis via the decreased proliferation of endothelial cells or accelerated senescence of endothelial progenitor cells, increasing endothelial permeability, decreasing TIMPs, and increasing MMP activity that may cause excess inflammation and fibrosis, and additionally by activating macrophages [98,99,100,101,102]. Of particular interest is miR-10a, whose decreased level is associated with the activation of the NF-κβ pathway, known to trigger miR-146a synthesis and release from the endothelium to cardiomyocytes [22,98]. Furthermore, miR21 may possess protective abilities by increasing NO synthesis, decreasing apoptosis, and strengthening vascular integrity [99,100,101].

### 4.3. Heat Shock Proteins

A new scientific direction in pathophysiology and a potential treatment strategy for PPCM concerns disturbances in the protein folding process with a potential role for heat shock proteins (Hsps), especially Hsp70, Hsp90, and small Hsp [102]. Hsps protect proper protein folding and prevent the accumulation of cellular toxic misfolded protein aggregations. However, in inflammatory and oxidative stress, Hsps may be secreted into the ECM, which can induce cardiomyocyte inflammation, hypertrophy, cell death, and fibrosis. High levels of Hsp70 have been found in HF; therefore, Hsp70 has been suggested as a potential HF biomarker and risk factor. To function properly, Hsp70 requires co-chaperons. The deficiency of one of them, BAG3, is associated with DCM. Moreover, mutations in the *BAG3* gene have been observed in PPCM [102].

Hsp90 possesses a cardioprotective function in case of hypoxia. However, Hsp90 was found to support Act signaling, playing a role in PPCM pathophysiology by increasing pathological cardiomyocyte hypertrophy. Therefore, Hsp90-inhibiting therapies may be beneficial for PPCM. On the other hand, small Hsps improve the survival of cardiomyopathy patients [102]. As presented in Figure 4, Hsps play an essential role in protecting the proteins taking part in the pathophysiology of PPCM: those acting in a cardioprotective manner, as well as those leading to PPCM. Thus, the potential beneficial role of drugs targeting Hsps in PPCM requires careful assessment. Of note, enhancing co-chaperone *BAG3* expression may offer cardioprotection in PPCM [102].

## 5. Therapy for Peripartum Cardiomyopathy

Currently, we are lacking a specifically targeted therapy for PPCM. Bromocriptine, the D2 receptor agonist that inhibits the secretion of PRL from the pituitary gland, in addition to standard HF pharmacotherapy, currently appears to be the most specific drug for PPCM [1,42]. The pathophysiology of PPCM and the effect of bromocriptine treatment were first validated on a STAT 3 CKO mouse model that developed PPCM during pregnancy and postpartum [21]. Mice with PPCM were characterized by increased cathepsin D levels, the presence of 16 kDa PRL, decreased levels of capillaries and cardiomyocytes in the heart, an increased level of MMP3, and fibrosis, which resulted in decreased survival correlated with an increased number of pregnancies [21].

**Figure 5:**
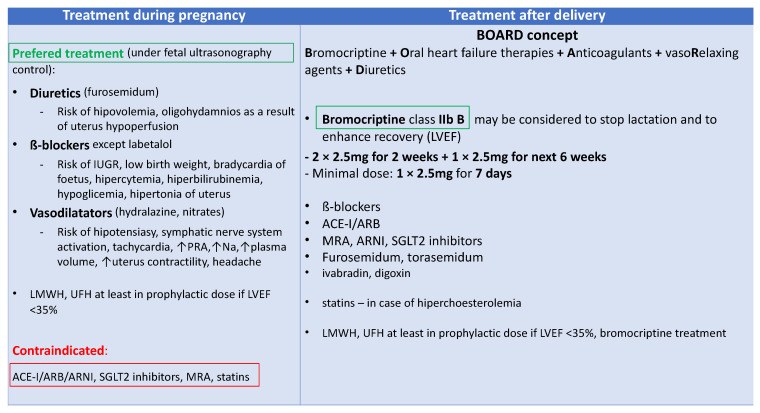
Peripartum cardiomyopathy (PPCM) treatment; ACE-I—angiotensin convertase enzyme inhibitor; ARB—angiotensin receptor blockers; ARNI—angiotensin receptor neprilysin inhibitor; IUGR—intrauterine growth retardation; LMWH—low molecular weight heparin; LVEF—left ventricular ejection fraction; MRA—mineral corticosteroid receptor agonists; Na—natrium; PRA—plasma renin activity; SGLT2—sodium-glucose cotransporter-2; UFH—unfractionated heparin.

Bromocriptine treatment was associated with an increased number of capillaries and cardiomyocytes in the heart, a decreased MMP3 level, and fibrosis [21]. The bromocriptine-treated mice had a normal shortening fraction and LV end-diastolic and end-systolic diameters in contrast to the untreated mice with PPCM [21]. Currently, PPCM treatment is based on the BOARD (Bromocriptine, Oral heart failure therapies, Anticoagulants, vaso-Relaxing agents, and Diuretics) concept that recommends using bromocriptine and anticoagulants on top of standard HF treatment (Figure 5) [60]. This concept originates from two randomized studies that suggested the beneficial role of bromocriptine treatment [42,103]. The outcome of the last randomized study highlights the necessity for further studies on bromocriptine, other pathophysiological aspects of the disease, and new drug targets.

### 5.1. New Biomarker-Based Therapies

Biomarkers associated with certain diseases may serve as a potential target for new therapies. The first reported target therapy for PPCM involved treating a mother with anti-miRNA-146a. One of the potential disadvantages of this treatment is that it may enable mothers to nurse neonates. However, studies on STAT 3 CKO mice have demonstrated that, in contrast to bromocriptine treatment, despite improvement in LV function, the LV remains dilated, suggesting that other pathological pathways have not been assessed with this treatment [22]. Other new therapies for microcirculatory dysfunction include the anti-sFlt-1 monoclonal antibody (mAb), which has been successful in the treatment of bronchopulmonary dysplasia in infants of mothers with preeclampsia in a rat model [104]. VEGF-modified RNA encoding VEGF (AZD-8601) was useful for the induction of therapeutic revascularization in the heart. In preclinical studies, it has been shown to regulate endothelial cells and cardiomyocyte survival and proliferation [105]. Pro-angiogenic therapy with recombinant VEGF was found to ameliorate PPCM [22]. However, VEGF treatment of PGC-1α HKO mice with sFlt-1-induced HF did not cause a full recovery from PPCM [22]. Therefore, treatment with anti-sFlt-1 mAb may improve results. The glucose-uptake-enhancing drug Perhexiline was found to decrease the cardiotoxic side effects of β-AR stimulation in CKO mice [106]. The cardioprotective property of this drug appears to be promising in patients with PPCM and cardiogenic shock when β-AR stimulation cannot be avoided [106].

The targets and biomarkers under investigation include proteins, such as Gal-3, proteoglycans, and miRNAs, which have been reviewed previously [107]. miRs that act as upstream regulators or downstream effectors of the fibrotic process may be useful in biomarker profiling for the identification of patients most likely to respond to the treatment with these agents. Some data demonstrate that fibrosis may be a reversible process. Therefore, as fibrosis is associated with an inferior outcome, more effort should be engaged in identifying therapeutic targets and developing new direct therapies [108,109]. New therapeutic targets in PPCM should include MPO and Gal-3. A novel, covalent, irreversible MPO inhibitor that decreases inflammation and improves microvascular function in preclinical models is currently being tested in a phase II clinical study (NCT03611153). The authors are investigating whether a single dose of 30 mg of AZD4831 given orally influences hemodynamic processes in patients with preserved LVEF ≥ 50% and with elevated filling pressures at rest or during exercise which can be assessed by pulmonary capillary wedge pressure during catheterization of the right heart. This is currently the most advanced clinical study on MPO inhibitors [110]. The available clinical data from phases I and II support further clinical development of AZD4831 for patients with HF with preserved ejection fraction. Anti-gal-3 therapy includes novel small-molecule gal-3 inhibitors, successful in the treatment of fibrosis in preclinical models, and modified citrus pectin multibranched polysaccharide, which ameliorated cardiac dysfunction, decreased myocardial injury, and decreased collagen deposition in rat HF models. It is worth mentioning that eplerenone and spironolactone downregulate gal-3 expression and therefore decrease the levels of collagen type I, collagen III, and TNF-α, preventing fibrosis after acute myocardial infarction in rats. Some molecules targeting Hsps are known to have a beneficial effect on improving HF. These include geranylgeranylacetone for increased Hsp70 expression, which was cardioprotective in cardiomyopathy models, as well as functional inhibitors that decrease the inflammatory effects of Hsps on cardiac tissue, such as an anti-Hsp70 antibody, polymixin B, colistin sulfate, and epigallocatechin-3-gallate [102].

### 5.2. Biomarker-Guided Therapy

Guiding HF therapy with biomarkers such as NT-proBNP and cardiac troponins can be helpful in clinical practice [5,8,9,10,11,12,13,14,15,16,17,18,19,20,21,22,23,24,25,26,27,28,29,30,31,32,33,34,35,36,37,38,39,40,41,42,43,44,45,46,47,48,49,50,51,52,53,54,55,56,57,58,59,60,61,62,63,64,65,66,67,68,69,70,71,72,73,74,75,76,77,78,79,80,81,82,83,84,85,86,87,88,89,90,91,92,93,94,95,96,97,98,99,100,101,102,103,104,105,106,107,108,109,110,111]. However, randomized trials on guiding therapy with natriuretic peptides have shown contradictory results. Some of them demonstrated the superiority of natriuretic peptide-guided HF treatment over traditional treatment based on clinical experience and guidelines. In these studies, the decrease in hospitalizations and mortality was lower in natriuretic peptide-guided therapy [112,113]. This was particularly true for patients ≤75 years of age [114]. However, some studies demonstrated no benefits from natriuretic peptide-guided therapy compared with clinically guided management, especially in older patients >60 years of age [115,116]. Metanalyses were found to have beneficial effects on natriuretic peptide-guided therapy according to a decrease in all-cause mortality compared with usual management, especially in younger patients. In addition, one demonstrated benefits such as a decrease in cardiovascular hospitalizations [117,118].

Studies on biomarker-managed therapy in PPCM are lacking. However, in patients with an improved LVEF in the six-month observation period, the levels of different biomarkers, including NT-proBNP, Fas/Apo1, IFN-γ, and prolactin, decreased more than in patients with no LVEF improvement [60]. A published example of one of our PPCM patients demonstrated that monitoring treatment with 23-kDa PRL may be beneficial in treating this disease, as an increase in PRL level after bromocriptine discontinuation was associated with the exacerbation of symptoms. Prolonged bromocriptine treatment for up to 12 months was particularly beneficial for this patient, with an increase in LVEF >50% [34].

## 6. Conclusions

In this review, the authors presented the current state of knowledge on pathophysiology, a broad range of biomarkers, and candidate biomarkers for PPCM, as well as on biomarker-targeted pharmacotherapy and biomarker-guided treatment. PPCM is rare; therefore, relatively few studies highlight the role of biomarkers in this disease, and it is not infrequent that some of these biomarkers have been investigated in only one study [30,41,59]. The etiology of PPCM is not fully understood and is complex, including a net of pathophysiological pathways of oxidative stress; inflammation; hormonal disturbances; dysfunction of endothelium, microcirculation, cardiomyocytes, and extracellular matrix; fibrosis; and genetic background. The disease is potentially life-threatening, affecting women of childbearing age. Even with LVEF recovery, there is still a high risk of relapse in subsequent pregnancies and the risk of death, irrespective of LVEF, in long-term observation remains extremely high.

Although nonspecific, NT-proBNP and BNP are currently the best diagnostic biomarkers in clinical practice, as they are easy to obtain, and there are many studies investigating their diagnostic and prognostic roles in HF of different origins. According to the current guidelines of the European Society of Cardiology on HF management, only NT-proBNP and BNP are recommended as diagnostic biomarkers [111]. Their assessment during treatment may be helpful in individual cases, although it is not recommended for guiding the treatment, and the role of biomarkers in treatment architecture is not clear [111]. 23-kDa PRL increases in physiological pregnancy and puerperium; therefore, it is not a candidate to be a diagnostic biomarker of PPCM; however, it may facilitate the management of treatment [35]. According to the current knowledge, 16-kDa PRL is the best candidate as a specific diagnostic biomarker for PPCM [21,42]. If every-day-use laboratory tests for 16-kDa PRL are developed, it can be additionally a more precise biomarker for treatment monitoring. The sFlt-1/PlGF ratio in postpartum PPCM patients was found to be lower than in healthy postpartum controls, at delivery, and in acute HF non-pregnant women. Therefore, the sFlt-1/PlGF ratio that can be obtained in everyday clinical practice, may serve as a diagnostic marker of PPCM after delivery and requires further validation [41]. Among the biomarkers associated with fibrosis, Gal-3 was found to be a diagnostic and prognostic biomarker associated with an increased risk of events and unrecovered LVEF in PPCM patients [30]. Higher PIIINP levels were associated with inferior prognoses in PPCM [30]. The PINP/PIIINP ratio may possess additional diagnostic and prognostic value, varying according to ethnicity [30]. Among the biomarkers associated with inflammation, the most specific PPCM diagnostic and prognostic biomarker was soluble Fas/Apo-1 [46]. However, its clinical usage is limited. Another biomarker, which was found to be diagnostic and prognostic, but not specific for PPCM, was IFN-γ. CRP and IL-6, although associated with PPCM’s pathological pathways, were not confirmed to possess diagnostic or prognostic potential. PAI-1 and TNF-α could serve as diagnostic biomarkers for PPCM. Additionally, PAI-1 is an interesting biomarker because its elevated level may be useful to foresee PPCM exacerbation in subsequent pregnancies [51]. Some biomarkers turned out to be linked to potential therapies, such as TNF-α, which is decreased by pentoxifylline and can decrease mortality in PPCM, or 16-kDa PRL, whose formation is inhibited by a blockade of 23-kDa PRL secretion by bromocriptine [42,73,103]. However, no normal ranges of these biomarkers for PPCM detection, prognosis, and management have been established so far.

More studies on new biomarkers for PPCM are required. MPO appears to be a more specific biomarker, and it can be obtained in everyday clinical practice. However, it has not been investigated in PPCM to date and new studies on its usage are required, especially in the context of the currently investigated MPO inhibitors [110]. Other potential directions of PPCM biomarker identification that may improve understanding of PPCM pathophysiology and cause new drug development include miRNAs, associated with endothelial and microcirculation homeostasis, and Hsps. The latest, just-published, work by Sliwa et al. showed that serum proteome profiling in PPCM patients may set new directions in pathophysiology assessment and the emergence of new specific biomarker candidates from different pathophysiological pathways, including immune response, inflammation, fibrosis, angiogenesis, and apoptosis, are significant in PPCM and that their roles need to be established [119].

### Take Home Messages

- Although rare, PPCM can be a life-threatening condition and may be difficult to diagnose. The etiology of PPCM is complex and remains unclear. The most important pathological pathways include antiangiogenic 16 kDa PRL, with bromocriptine being the most specific PPCM treatment to date.
- There is a need to explore new pathophysiological pathways concerning endothelial miRs, ECM fibrosis, and cardiac tissue proteostasis, as well as to identify new drug targets to improve patient outcomes.
- A broad range of drug molecules requires further testing in PPCM, particularly concerning the safety of the MPO inhibitor, which is the most advanced in clinical HF studies to date.
